# Methylartist: tools for visualizing modified bases from nanopore sequence data

**DOI:** 10.1093/bioinformatics/btac292

**Published:** 2022-04-28

**Authors:** Seth W Cheetham, Michaela Kindlova, Adam D Ewing

**Affiliations:** Australian Institute for Bioengineering and Nanotechnology, University of Queensland, St Lucia, Australia; Mater Research Institute—University of Queensland, Translational Research Institute, Woolloongabba, QLD 4102, Australia; Australian Institute for Bioengineering and Nanotechnology, University of Queensland, St Lucia, Australia

## Abstract

**Summary:**

Methylartist is a consolidated suite of tools for processing, visualizing and analysing nanopore-derived modified base calls. All detectable methylation types (e.g. 5mCpG, 5hmC, 6mA) are supported, enabling integrated study of base pairs when modified naturally or as part of an experimental protocol.

**Availability and implementation:**

Methylartist is implemented in Python and is installable via PyPI and bioconda. Source code and test data are available at https://github.com/adamewing/methylartist.

**Supplementary information:**

Supplementary data are available at *Bioinformatics* online.

## 1 Introduction

Covalent modification of nucleobases is an important component of genomic regulatory regimes across all domains of life (Blow *et al.*, 2016; Couturier and Lindås, 2018; Zemach *et al.*, 2010) and is harnessed by genomic footprinting assays, including DamID (van Steensel and Henikoff, 2000), SMAC-seq (Shipony *et al.*, 2020) and NOMe-seq (Lee *et al.*, 2020). Nanopore sequencing offers comprehensive assessment of base modifications from arbitrarily long sequence reads through analysis of electrical current profiles, generally with machine learning models trained to discriminate between modified and unmodified bases (Simpson *et al.*, 2017). An increasing number of computational tools have been developed or enhanced for calling modified bases (Yuen *et al.*, 2021), including nanopolish (Simpson *et al.*, 2017), DeepSignal (Ni *et al.*, 2019), megalodon (Oxford Nanopore Technologies), guppy (Oxford Nanopore Technologies) and Nanocompore (Leger *et al.*, 2021).

## 2 Materials and methods

Experimental and computational methods are detailed in Supplementary Material, as well as a discussion of other methods for visualizing nanopore-derived base modification data.

Methylartist is implemented in Python using SQLite (Hipp, 2020), matplotlib (Hunter, 2007), seaborn (Waskom, 2021), numpy (Harris *et al.*, 2020), scipy (Virtanen *et al.*, 2020), pandas (McKinney, 2010), scikit-bio (The Scikit-Bio Development Team, 2020), pysam (Li *et al.*, 2009) (https://github.com/pysam-developers/pysam), bx-python (https://github.com/bxlab/bx-python) and the ONT fast5 API (https://github.com/nanoporetech/ont_fast5_api). Methylartist is available at https://github.com/adamewing/methylartist, via pip install methylartist and via conda install -c bioconda methylartist.

Further examples with sample data are available from the methylartist testing repository at https://github.com/adamewing/methylartist-tests.

Command-line arguments to methylartist for all figures presented in this manuscript are available in Supplementary Materials. Additional examples are available at https://github.com/adamewing/methylartist.

## 3 Results and discussion

Methylartist offers novel and useful visualization outputs complementary to those available through extant nanopore visualization tools (De Coster *et al.*, 2020; Pryszcz and Novoa, 2021; Su et al., 2021). Methylartist supports arbitrary modifications, which has utility for identification of modified bases in assay-specific contexts including GpC methylation (NOMe-seq), and 6mA (SMAC-seq, DamID in a 5ʹ-GATC-3ʹ context, as well as native RNA base modifications). With few exceptions (Begik *et al.*, 2021; Li *et al.*, 2021), most currently available models for calling modified bases involve some form of methylation or hydroxymethylation, so modifications will be referred to collectively as ‘methylation’, without loss of generality.

**Fig. 1.**
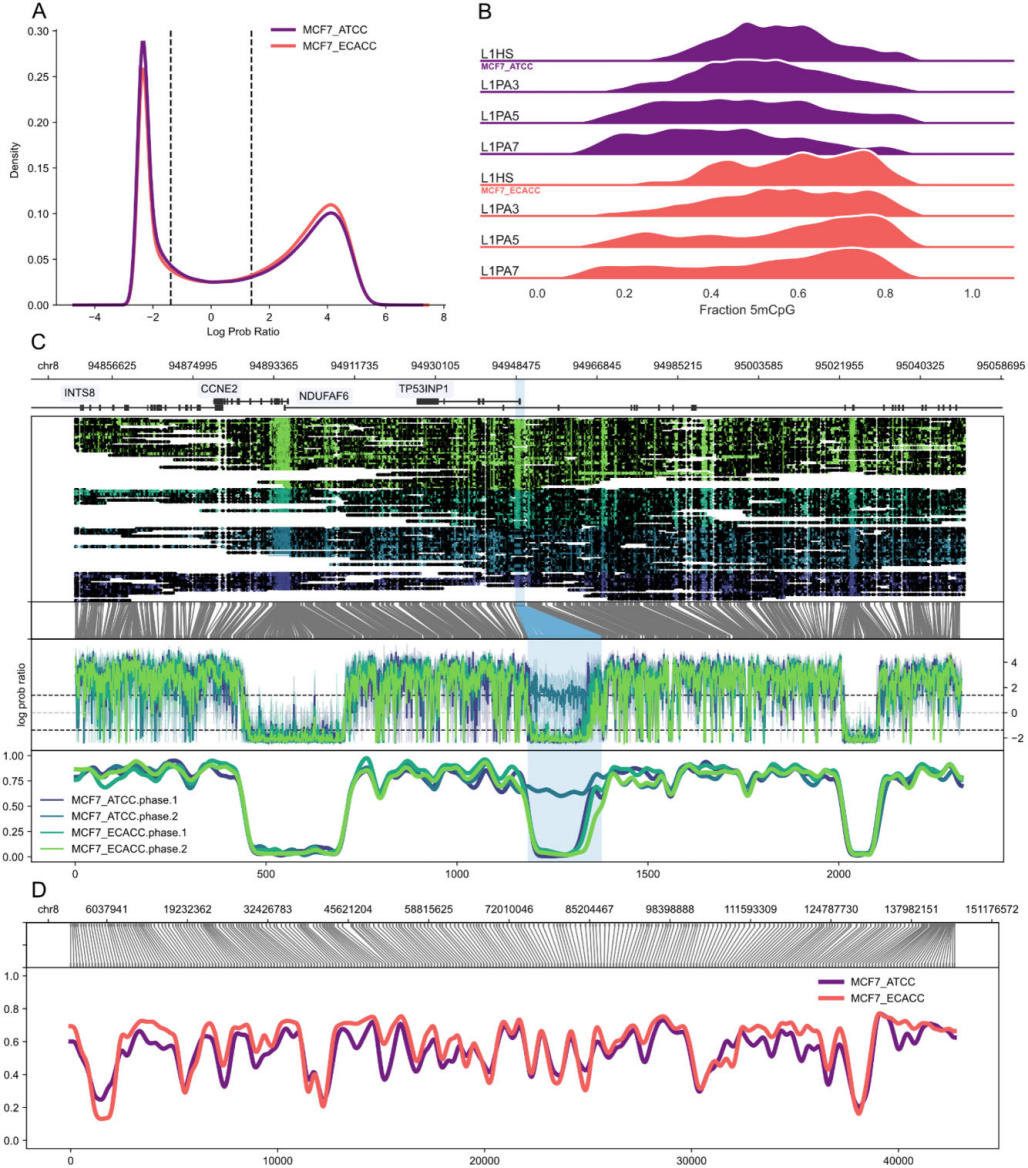
Examples of methylartist output. (**A**) Plot of the distribution (kernel density estimate) of methylation scores (log probability ratio) from megalodon for MCF7 cultivars. The dashed vertical lines indicate the cutoffs for calling a CpG unmethylated (left cutoff) or methylated (right cutoff). (**B**) Ridge plots output by the segplot tool for selected human LINE-1 families, selected here for aesthetic demonstration purposes. (**C**) Allele-specific methylation profiles (locus plot) for TP53INP1. For locus plots, the panels show the following information from top to bottom: genes (exons as boxes, introns as connecting lines) with optional labels, read alignments grouped and coloured by sample with methylation motifs (CpG) marked as open or closed dots, translation from genome coordinate space into a reduced modified base space (in these cases, CG dinucleotides), a ‘raw’ plot of the methylated base statistic (in this case, log probability ratios) and finally a smoothed plot of the methylation profile. This plot also demonstrates the use of highlights, which can be used to indicate regions of interest (in this case, selected CpG islands). (**D**) Demonstration of a larger scale methylartist region plot comprising all of human chromosome 8. The content of the plot is as described for panel (C) but without the read alignment or raw statistic plots. Coordinates across the bottom refer to methylation bins used in the smoothed methylation profile plot

Modified bases are called from signal-level data using a variety of software tools with an appropriate basecalling model. Methylartist supports input from BAM files with base modification tags that conform to the SAM Optional Fields Specification. Alternatively, tabular per-read modified base calls can be imported from a variety of formats including megalodon (via the db-megalodon function), nanopolish (db-nanopolish), guppy (db-guppy) and any tabular format that outputs a read name, base position and a probability of base modification (db-custom). For imported tabular data, methylartist includes a method ‘methylartist scoredist’ to plot the distribution of base modification statistics which is useful in quality control and ensuring successful data import (Fig. 1A). Modification and non-modification call cutoffs can be adjusted via ‘methylartist adjustcutoffs’. To demonstrate the capabilities of methylartist, we sequenced MCF-7 cells sourced from ATCC and from ECACC on the Oxford Nanopore Technologies PromethION platform. MCF-7 is a widely studied breast cancer cell line with sub-lines often expressing divergent cellular phenotypes (Ben-David *et al.*, 2018; Comşa *et al.*, 2015). We anticipated that sourcing cells originating from different repositories would yield locally different methylation profiles suitable for demonstration purposes.

The command ‘methylartist segmeth’ aggregates methylation calls over segments into a table of tab-separated values, useful for comparing whole-genome methylation or methylation over various annotations such as promoters, enhancers or transposable element families. The resulting table is useful on its own or as input to ‘methylartist segplot’ or ‘methylartist composite’. Category-based methylation data aggregated with ‘segmeth’ can be plotted as strip plots, violin plots or ridge plots using the ‘segplot’ command (Fig. 1B).

Locus- or region-specific plots can be created in two ways, depending on the size of the window. For smaller sub-megabase regions, ‘methylartist locus’ will generate plots similar to the example in Figure 1C, which shows haplotype-specific methylation profiles for the TP53INP1 locus in the two MCF7 cultivars. Examples of non-phased methylation profiles are included as Supplementary Figure S1, and a further example of the known paternally imprinted differentially methylated region for PEG3 is included as Supplementary Figure S2. These locus plots, from top to bottom, include an optional track showing genes, methylation calls relative to aligned read positions, a translation from genome space into a modified base space consisting only of instances of the methylated motif, a plot of the methylation statistic (e.g. log likelihood ratio) and a smoothed sliding-window plot showing methylation fraction across the region. As shown in Figure 1C, the ‘locus’ plotting function supports separating methylation profiles by phase, if the .bam files are first phased via WhatsHap (Patterson *et al.*, 2015) or another tool to add the ‘PS’ and ‘HP’ tags. For larger regions, roughly greater than a megabase, ‘methylartist region’ is recommended to aggregate methylation calls into bins, which are normalized for occurrences of the methylation motif. Region plots can span an entire chromosome efficiently (Fig. 1D). Both locus and region plots support an extensive set of parameters controlling dimensions, colour selection, highlighting, smoothing parameters and panel ratios and visibility. All plots allow visualization of modified base profiles beyond CpG methylation, and examples using 6mA footprinting (SMAC-seq) are included in Supplementary Figures S3–S5.

In order to facilitate the study of methylation patterns across families of highly duplicated sequences such as transposable elements (Ewing *et al.*, 2020), methylartist supports a ‘composite’ methylation plot, which aligns each instance of a repeat element family to a user-supplied consensus sequence and shows the methylation profile of a user-defined number of individual elements (Supplementary Fig. S6). Finally, the ‘wgmeth’ tool in methylartist can output bedMethyl files and files suitable for input to DSS, a package for assessing differential methylation (Park and Wu, 2016).

## 4 Conclusion

Methylartist has substantial utility as a tool for plotting and analysing nanopore-derived modified base data. It is an accessible augmentation to the available tools for analysis and visualization of nanopore-derived methylation data, including the non-CpG modification motifs used in chromatin footprinting assays. Functionality will be expanded and updated in the future as new use cases arise and as methods for analysis of nanopore data continue to evolve.

## Supplementary Material

btac292_Supplementary_Data
