# Supplementary material for: Methylartist: tools for visualizing modified bases from nanopore sequence data
Source: Bioinformatics. 2022 Apr 28;38(11):3109–12. doi: 10.1093/bioinformatics/btac292 (PMC9154218; doi:10.1093/bioinformatics/btac292)
Supplement: btac292_Supplementary_Data [file btac292_supplementary_data.zip › Methylartist Supplemental Material.pdf]

**Supplemental Material for "Methylartist: Tools for Visualising Modified Bases from Nanopore Sequence Data"**

## Methods

Cell culture: MCF-7 cells (ATCC, ECACC) were grown to 60-80% confluency in high-glucose Dulbecco's Modified Eagle Medium (DMEM, Life Technologies) supplemented with 10% heat-inactivated Fetal Bovine Serum (Life Technologies), 2mM L-Glutamine (Life Technologies) and 100U/mL Penicillin-Streptomycin solution (Life Technologies). Cells were washed with Dulbecco's Phosphate Buffered Saline (DPBS, Life Technologies), lifted with Trypsin 0.25% EDTA (Life Technologies), pelleted by centrifugation, and washed again with DPBS.

Long-read PromethION sequencing: Genomic DNA was isolated using a Circulomics Big DNA Tissue Kit. Both the high molecular weight (HMW) and ultra-high molecular weight (UHMW) protocol was carried out for each cultivar (ATCC, ECACC) according to the manufacturer's instructions for a total of 4 PromethION flow cells (Supplemental Table 1). Due to high DNA viscosity the UHMW protocol was modified to include vortexing after addition of CLE3 digestion buffer and incubation at 37°C instead of RT. For simplicity and for demonstration purposes replicates were combined across cultivars to yield one high-depth ATCC sample and one high-depth ECACC sample.

SMAC-seq: For both experimental conditions tested, 1x10^6 MCF-7 cells (ATCC) were resuspended in 500 µl of ice-cold Cell Fractionation Buffer (Abcam) and incubated for 10 minutes on ice. Nuclei were pelleted by centrifugation for 3 min at 500g, then resuspended in 200 µl of ice-cold Nuclei Wash Buffer (10 mM Tris pH7.4, 10mM NaCl, 3mM MgCl2, 0.1 mM EDTA). The nuclei were then pelleted by centrifugation for 3 min at 500g and resuspended in EcoGII reaction buffer (1X NEB CutSmart Buffer, 0.3 M sucrose). 200U of EcoGII and 0.6 mM SAM were added and nuclei were incubated either at 37°C for 10 min (condition one) or incubated at 37°C with 1000 rpm agitation for 15 min, with SAM replenished at 7.5 min (condition two). DNA was extracted with the Monarch® Genomic DNA Purification Kit (NEB) according to manufacturer's instructions with mixing by inversion instead of vortexing. 1 µg of Genomic DNA was prepared for Nanopore sequencing using the Ligation Sequencing Kit (Nanopore LSK110). Samples were sequenced for 72 hr on an r9.4 MinION flowcell on the Nanopore MinION Mk1C.

Statistics for sequencing runs are included in Supplemental Table 1.

Read alignment and variant calling: Nanopore reads were aligned to hg38 via minimap2 2.17(Li, 2018) with parameters -a -x map-ont --cs:long --MD. Illumina reads were mapped to hg38 via bwa mem2 2.0pre2 (sse4.1) with default parameters and duplicate reads were marked via the MarkDuplicates tool in Picard 2.23.8(http://broadinstitute.github.io/picard).

Phasing: To inform phasing, variants were detected in the MCF-7 Illumina data using HaplotypeCaller in GATK 4.1.9.0 (DePristo *et al.*, 2011). The resulting VCF was phased

using whatshap 1.0(Patterson *et al.*, 2015) and aligned nanopore reads. Nanopore reads were then tagged with haplotypes using the 'haplotag' function of whatshap.

Methylation calling: Basecalling along with modified base calls was done using megalodon 2.2.9 with guppy 4.4.0 using the res_dna_r941_prom_modbases_5mC_v001 model for 5mCG detection (PromethION), or the res_dna_r941_min_modbases-all-context_v001 model for 6mA detection (MinION).

### SMAC-seq

To demonstrate analysis and plotting of non-CpG methylation in a relevant context, we carried out a version of SMAC-seq (Shipony *et al.*, 2020), in which nuclei are treated with EcoGII (Murray *et al.*, 2018) to enrich accessible chromatin with 6mA modifications. We analysed the SMAC-seq data with megalodon using the "res_dna_r941_min_modbases-all-context_v001" model from the rerio repository (https://github.com/nanoporetech/rerio), created a methylartist database via "db-megalodon" and identified loci from the eukaryotic promoter database (EPD) (Dreos *et al.*, 2017), with high 6mA relative to unmodified adenine using the "segmeth" utility in methylartist. In general, we see higher apparent 6mA methylation in regions defined by the Eukaryotic Promoter Database as compared to 50k size-matched regions of the genome drawn at random (Supplemental Figure 2a) which, as expected, is the opposite of 5mCG methylation between promoters and the genome as a whole (Supplemental Figure 2b). These loci were plotted en masse via the "locus" tool and screened visually, examples with corresponding CpG methylation plot are shown in Supplemental Figures 3 and 4. Methylartist supports settings to improve visualisation of data where the expected distribution is a series of peaks, including the ability to limit inclusion of reads with an unusually high fraction of methylated bases (--maxfrac), and the option to skip sites below a threshold of methylated + unmethylated call coverage (--mincalls).

### Survey of selected comparable tools

Perhaps the most widely-used methods for visualising nanopore-derived modified base calls are genome browsers including IGV (Robinson *et al.*, 2011) and JBrowse2 (Buels *et al.*, 2016). These tools allow for the display of modified base calls over read plots similar to the read alignment panel output by "methylartist locus", but are better suited for interactive use. This functionality can be augmented by NanoMethPhase (Akbari *et al.*, 2021) which facilitates viewing phased methylation data in a genome browser, along with potentially improved phasing. An example methylartist workflow might involve identifying regions of interest via a genome browser followed by plotting with "methylartist locus" to generate visually appealing output for presentation or publication.

As a number of tools offering overlapping sets of features exist for plotting modified base call data, we surveyed the salient features of comparable tools aimed at nanopore-derived modified base calls, with results summarised in Supplemental Table 2. Within each feature category there is a wide degree of variation as to the visual style of the resulting plots. In the

table below, “Per-read” refers to a genome-browser-esque view that includes real alignments and modified base call sites. “Aggregate” refers to the ability to plot multiple sites in a single window (e.g. Supplemental Figure 6). “Phasing” refers to an ability to take haplotagged data into account, “MDS” refers to a multidimensional scaling or similar reduction of dimensionality across samples, “Segment” refers to plotting modified base statistics over segments with different annotations and “Score” refers to the ability to plot the distribution of raw baseball scores as per Figure 1a. This table does not capture all features of each tool given that some are hard to categorise e.g. the “spaghetti plots" available through nanomethviz are somewhat comparable to the smoothed methylation plots offered by the locus and region tools in methylartist, but offer different levels of information. Some tools are aimed at data processing and analysis versus visualisation (e.g. modPhred) and so offer useful functionality not captured here.

| | Per-read | Aggregate | Phasing | MDS | Segment | Score |
|---|---|---|---|---|---|---|
| methylartist | Yes | Yes | Yes | No | Yes | Yes |
| nanomethviz | Yes | Yes | Yes | Yes | Yes | No |
| Methplotlib | Yes | No | Yes | Yes | No | No |
| modPhred | Yes (IGV) | No | No | No | No | Yes |

Supplemental Table 2: Comparison of selected features across selected visualisation tools.

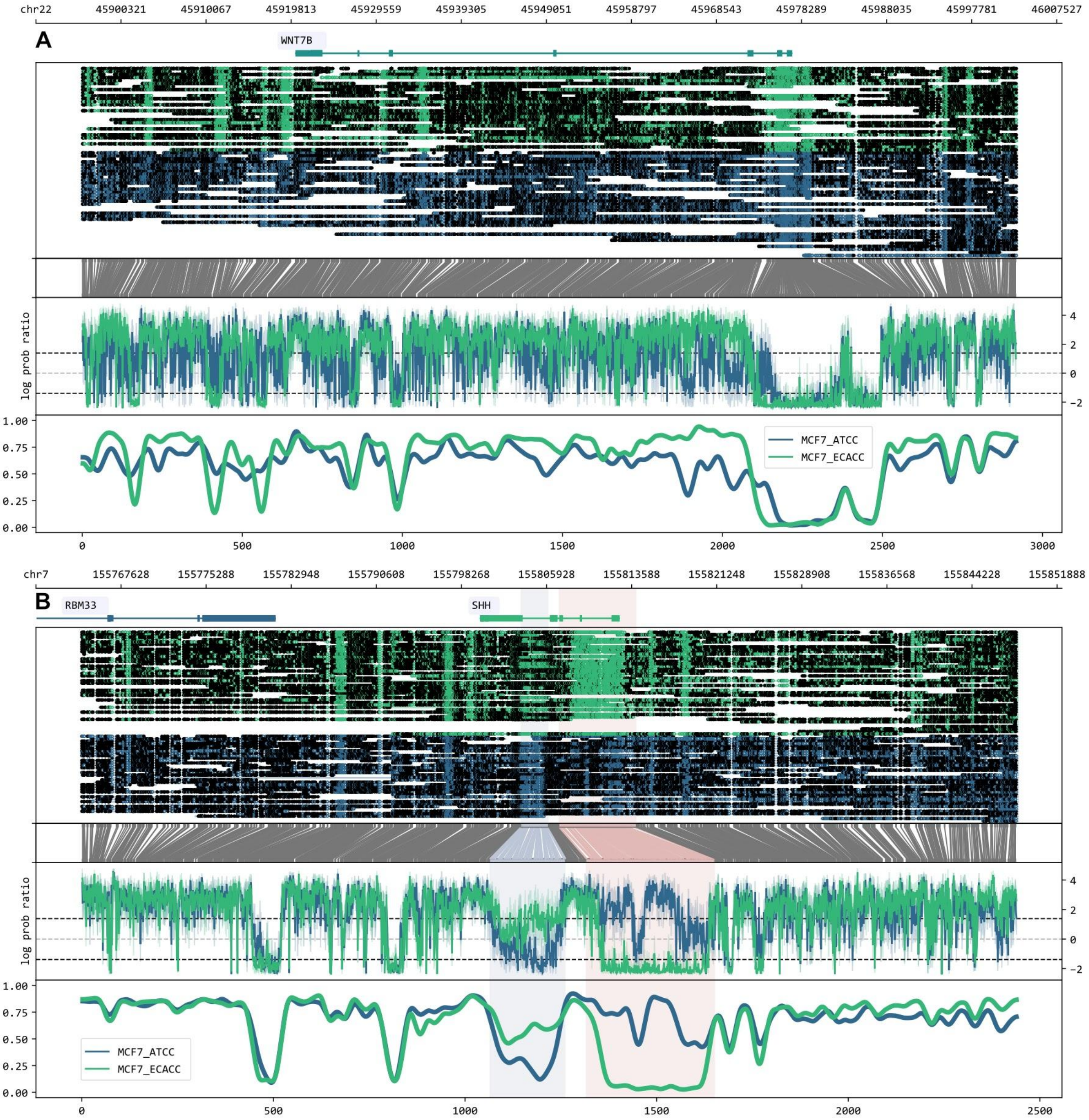


**Supplemental Figure 1: Methylartist locus plots** showing methylation profiles for WNT7B **(A)**, and SHH **(B)**. For each plot, the panels show the following information from top to bottom: genes (exons as boxes, introns as connecting lines) with optional labels, read alignments grouped and coloured by sample with methylation motifs (CpG) marked as open or closed dots, translation from genome coordinate space into a reduced modified base space (in these cases, CG dinucleotides), a “raw” plot of the methylated base statistic (in this case, log probability ratios), and finally a smoothed plot of the methylation profile. Plot **(B)** demonstrates the use of highlights, which can be used to indicate regions of interest (in these cases, selected CpG islands).

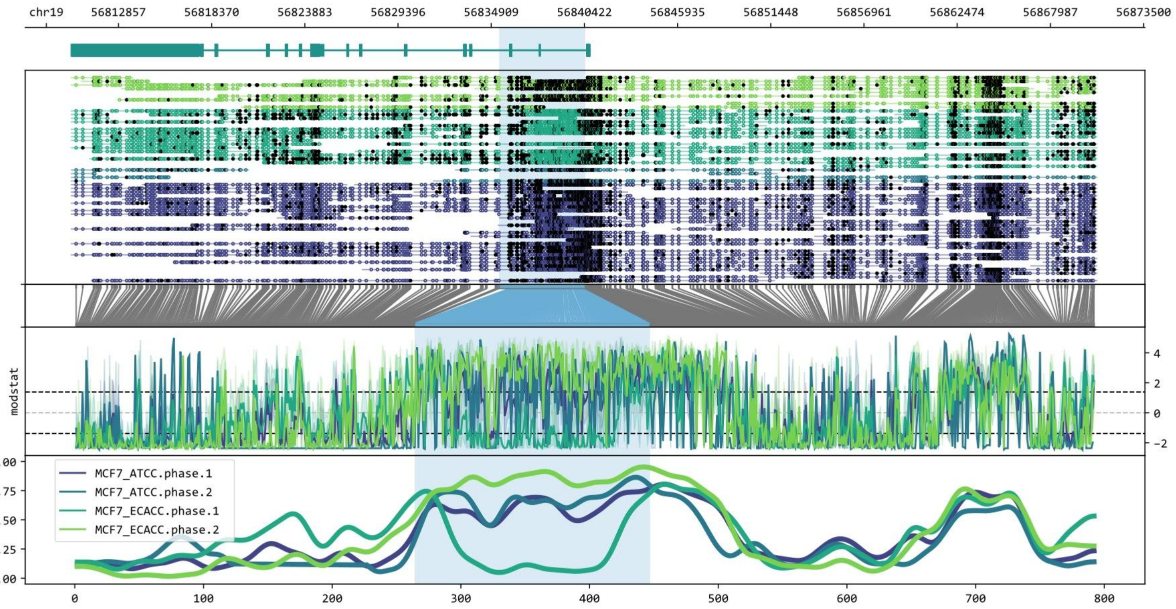


**Supplemental Figure 2:** Similar to Figure 1c, this shows the phased methylation profile of the known paternally imprinted locus PEG3.

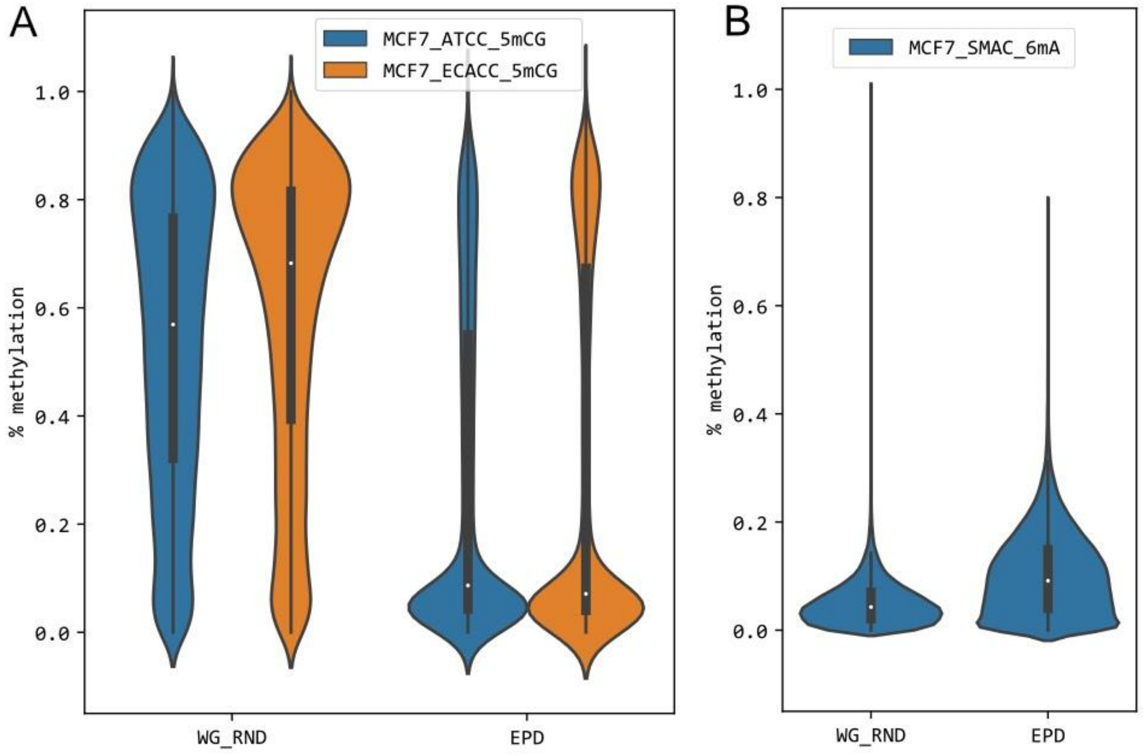


**Supplemental Figure 3:** Violin plots from methylartist segplot showing **(a)** the overall 5mCG distribution among 50000 random 590-bp segments of the genome (WG_RND) compared to a +/- 250bp window around eukaryotic promoter database (EPD) promoter annotations, which are 90bp. Panel **(b)** is similar to **(a)**, showing 6mA instead of 5mCG from SMAC-seq on ATCC MCF-7 cells.

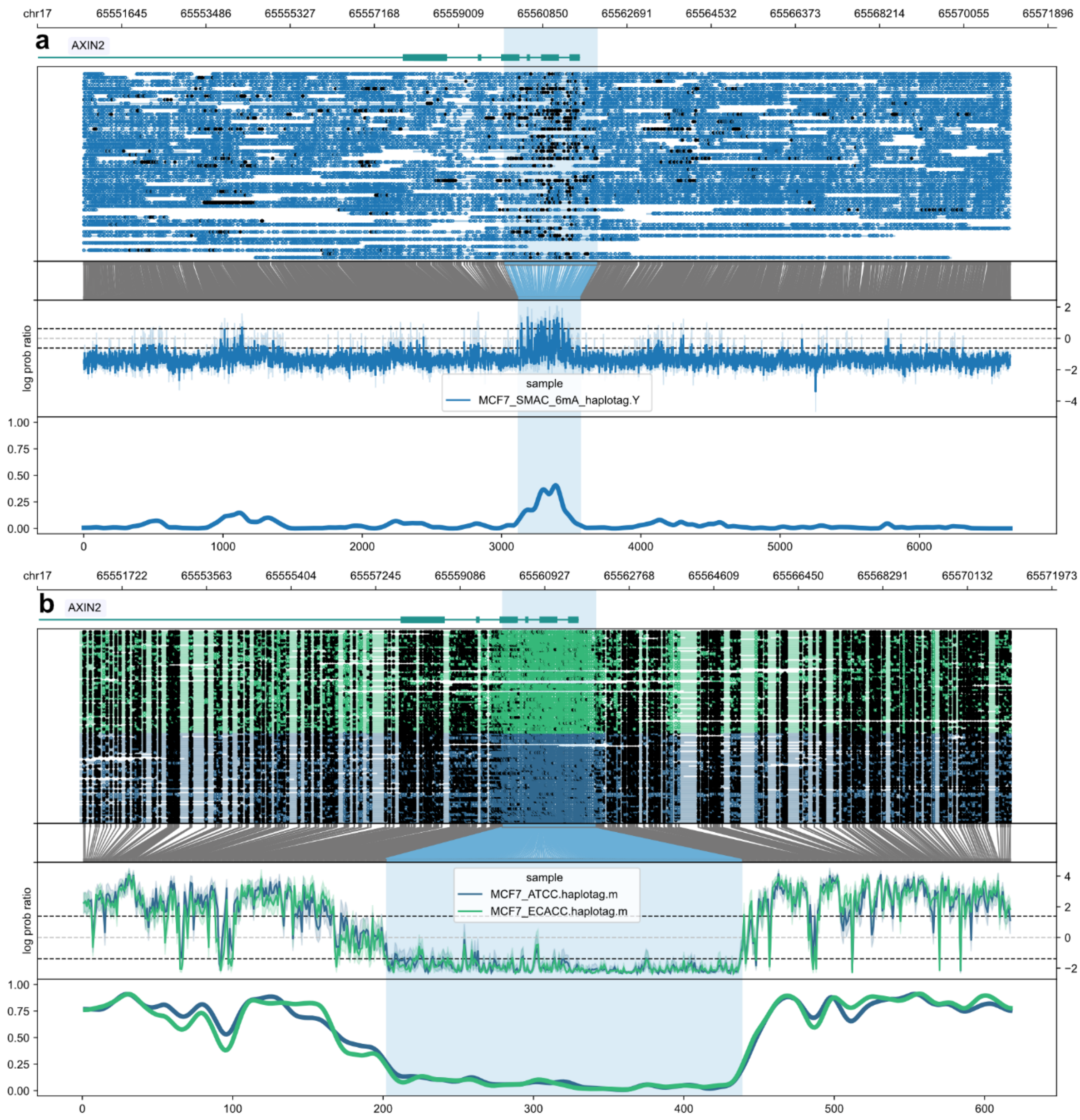


**Supplemental Figure 4: Example plot incorporating non-CpG methylation** comparing the 6mA profile for an AXIN2 promoter from SMAC-seq on ATCC MCF-7 cells **(A)** with the 5mCG methylation profile from both MCF-7 cultivars used in this study **(B)**. Plots are as described in Figure 1, substituting A for CG in the modified base space in plot (A). The highlighted region, which is the same in both plots, corresponds to increased 6mA indicating accessible chromatin in plot **(A)** and decreased 5mCG consistent with regulatory elements in plot **(B)**.

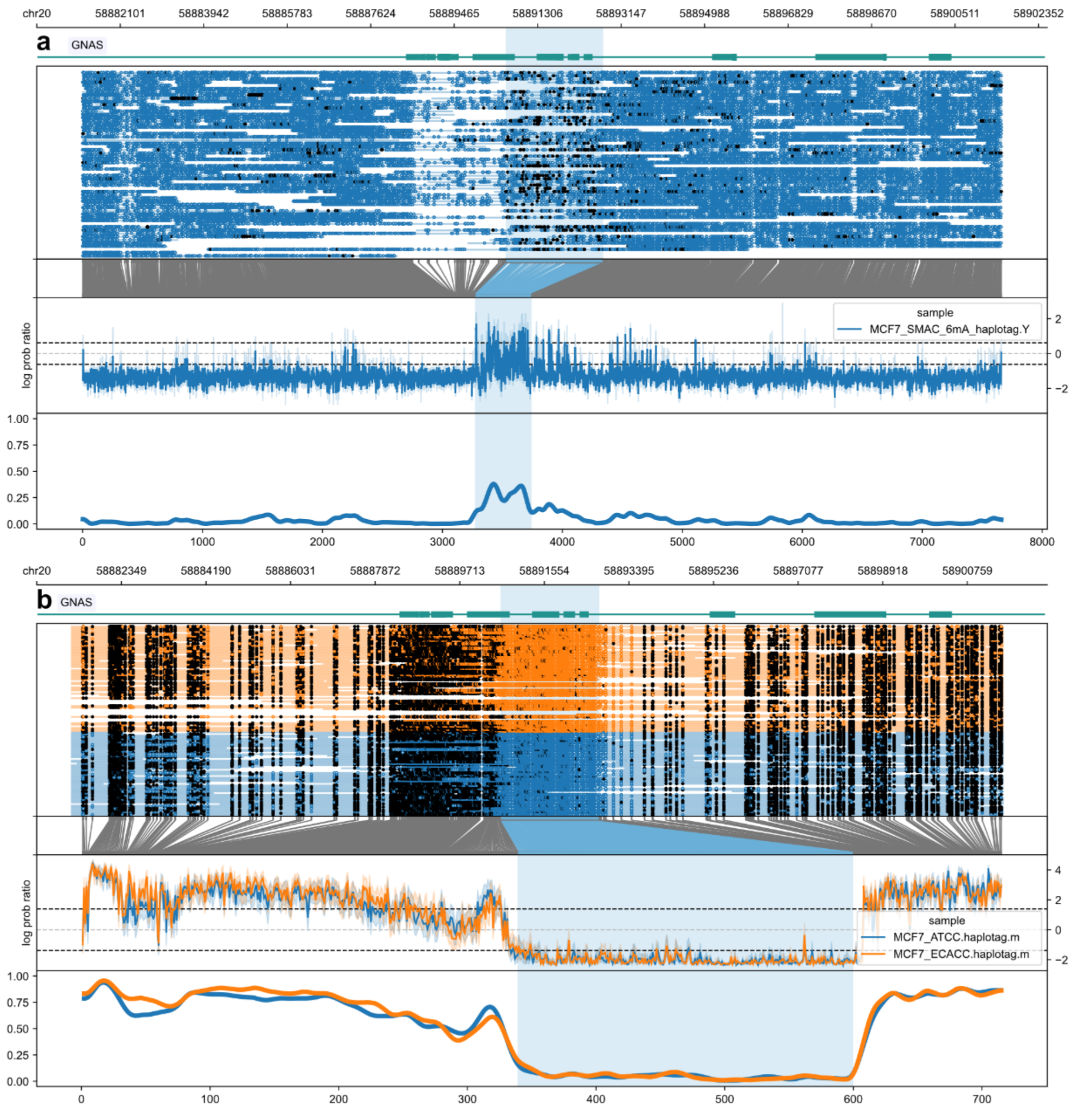


**Supplemental Figure 5:** Analogous to Supplemental Figure 4 but for a regulatory element of the GNAS gene.

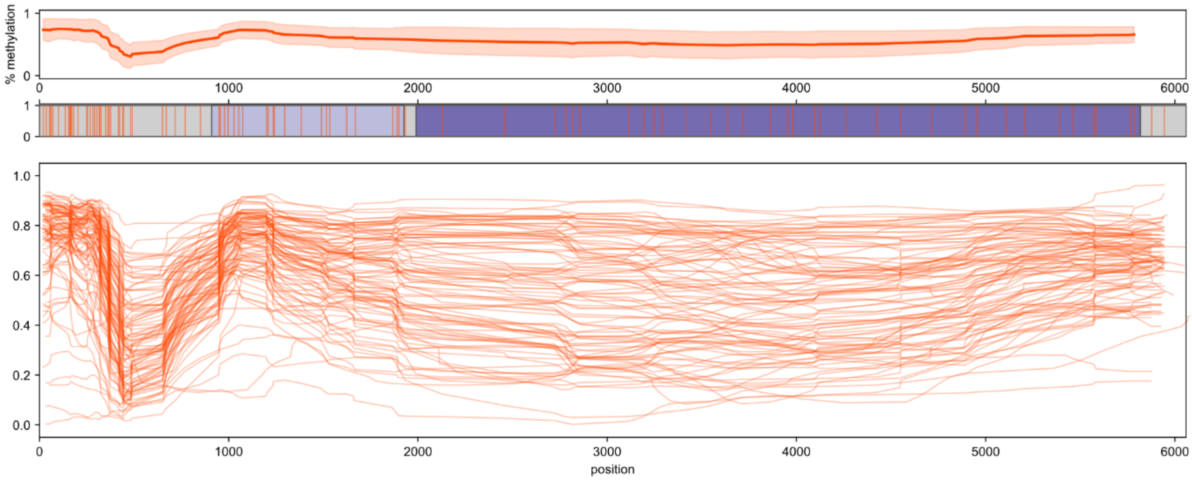


**Supplemental Figure 6: Repetitive element composite methylation profiles.** Methylation (5mCG) profiles of full length (~6000bp) human-specific LINE-1 elements in ECACC MCF-7 cells using the methylartist composite function. From top to bottom the elements of this plot include an indication of the average methylation level across the L1 consensus element, a plot indicating features (ORF1, ORF2) of the L1 consensus sequence (controlled through a table input to methylartist, orange vertical lines = CpGs), and a plot of the methylation profiles of 300 individual full-length L1 elements, randomly selected.

**Supplementary Material: Command-line arguments**

For data loading examples (nanopolish, megalodon, guppy, and custom+), please see the github README at https://github.com/adamewing/methylartist. Timing information is relative to a Dell Poweredge R7515 Server: AMD 7702P (2Ghz / 64 Cores / 128 Threads), 512 GB RAM (3200MT/s RDIMMs) with SAS Mixed-use SSDs (12 Gbps), NVidia Tesla T4 16GB.

**Score distribution (scoredist):**

Figure 1a:

methylartist scoredist -d MCF7_ATCC.megalodon.db,MCF7_ECACC.megalodon.db -m m --palette magma –lw 3 --svg

Time: 7m7s

**Ridge plot (segmeth, segplot):**

Figure 1b:

methylartist segmeth -d MCF7_data_megalodon.txt -i L1_FL.bed -p 32

Time: 0m44s

methylartist segplot -s L1_FL.MCF7_data_megalodon.segmeth.tsv -g --palette magma -c L1HS,L1PA3,L1PA5,L1PA7 --svg

Time: 0m4s

**Violin plots (segmeth, segplot)**

Supplemental Figure 3a:

methylartist segmeth -d MCF7_data_megalodon.txt -i epdNewHuman006.hg38.pad250.rename.hg38.590bp.50krnd.bed -p 32

Time: 9m44s

methylartist segplot -s epdNewHuman006.hg38.pad250.rename.hg38.590bp.50krnd.MCF7_data_megalodon.segmeth.tsv -v --svg

Time: 0m19s

Supplemental Figure 3b:

methylartist segmeth -d MCF7_SMAC_data.txt -i epdNewHuman006.hg38.pad250.rename.hg38.590bp.50krnd.bed -p 32 --max_read_density 0.3

methylartist segplot -s epdNewHuman006.hg38.pad250.rename.hg38.590bp.50krnd.MCF7_SMAC_data.segmeth.tsv -v --svg

**Locus Plots (locus)**

Wnt7b (Supplemental Figure 1a):

methylartist locus -d MCF7_data_megalodon.txt -i chr22:45895804-46003016 -g Homo_sapiens.GRCh38.97.chr.sorted.gtf.gz --genes WNT7B --labelgenes --samplepalette viridis --statname log\ prob\ ratio --svg

Time: 5m29s

SHH (Supplemental Figure 1b):

methylartist locus -d MCF7_data_megalodon.txt -i chr7:155764089-155848355 -l 155803598-155806098,155807024-155814024 -g Homo_sapiens.GRCh38.97.chr.sorted.gtf.gz --labelgenes --samplepalette viridis --highlightpalette vlag --statname log\ prob\ ratio --svg

Time: 5m33s

**Phased Locus Plots (locus --phased)**

TP53INP1 (Figure 1c):

methylartist locus -d MCF7_data_megalodon.txt -i chr8:94848291-95050367 -l 94948291-94950367 -g Homo_sapiens.GRCh38.97.chr.sorted.gtf.gz --samplepalette viridis --maskcutoff 1 --labelgenes --phased --genes INTS8,CCNE2,TP53INP1,NDUFAF6 --statname log\ prob\ ratio --svg

Time: 9m8s

PEG3 (Supplemental Figure 2):

methylartist locus -d MCF7_data_megalodon.txt -i chr19:56810076-56870725 -l 56835376-56840476 -g Homo_sapiens.GRCh38.97.chr.sorted.gtf.gz --genes PEG3 --slidingwindowsize 20 -s 36 --samplepalette viridis --phased --maskcutoff 0 --svg

Time: 1m40s

**Region Plots (region)**

Figure 1d:

methylartist region -d MCF7_data_megalodon.txt -p 32 -r /home/data/ref/hg38/Homo_sapiens_assembly38.fasta -n CG -i chr8:1-145138636 --svg

Time: 19m58s

**Composite Plots (composite)**

Supplemental Figure 6:

methylartist composite -b MCF7_ECACC.haplotag.bam -m MCF7_ECACC.megalodon.db --sample MCF7_ECACC.haplotag_m -s L1HS.MCF7_data_megalodon.excl_ambig.segmeth.tsv -f L1HS -r Homo_sapiens_assembly38.fasta -t L1.3.fa -p 32 --blocks L1.3.highlights.bed --plotmean --svg

Time: 0m34s

**SMAC-seq Locus Plots (locus)**

AXIN-2 5mCpG (Supplemental Figure 4a):

methylartist locus -d MCF7_data_megalodon.txt -i chr17:65550815-65571075 -l 65560000-65562050 -g Homo_sapiens.GRCh38.97.chr.sorted.gtf.gz --samplepalette viridis --genes AXIN2 --labelgenes --statname log\ prob\ ratio --svg

AXIN-2 6mA (Supplemental Figure 4b):

methylartist locus -d MCF7_SMAC_data.txt -i chr17:65550815-65571075 -l 65560000-65562050 -g Homo_sapiens.GRCh38.97.chr.sorted.gtf.gz --genes AXIN2 --labelgenes --max_read_density 0.3 --maskcutoff 0 --mincalls 4 --statname log\ prob\ ratio --svg

GNAS 5mCpG (Supplemental Figure 5a):

methylartist locus -d MCF7_data_megalodon.txt -i chr20:58881271-58901531 -l 58890600-58892750 -g Homo_sapiens.GRCh38.97.chr.sorted.gtf.gz --genes GNAS --labelgenes --statname log\ prob\ ratio --svg

GNAS 6mA (Supplemental Figure 5b):

methylartist locus -d MCF7_SMAC_data.txt -i chr20:58881271-58901531 -l 58890600-58892750 -g Homo_sapiens.GRCh38.97.chr.sorted.gtf.gz --genes GNAS

```
--labelgenes --max_read_density 0.3 --maskcutoff 0 --mincalls 4 --statname log\ prob\ ratio
--svg
```

**Phasing (WhatsHap):**

1. Phase the VCF derived from Illumina sequencing and GATK:

```
whatshap phase -o MCF7.haplotypecaller.recal.annotated.known.phased.vcf
--reference=Homo_sapiens_assembly38.fasta --ignore-read-groups
MCF7.haplotypecaller.recal.annotated.known.vcf MCF7_ATCC.bam MCF7_ECACC.bam
```

2. Tag haplotypes in Nanopore data:

```
whatshap haplotag -r /home/data/ref/hg38/Homo_sapiens_assembly38.fasta
--ignore-read-groups --tag-supplementary -o MCF7_ECACC.haplotag.bam
MCF7.haplotypecaller.recal.annotated.known.phased.vcf.gz MCF7_ECACC.bam
```

```
whatshap haplotag -r /home/data/ref/hg38/Homo_sapiens_assembly38.fasta
--ignore-read-groups --tag-supplementary -o MCF7_ATCC.haplotag.bam
MCF7.haplotypecaller.recal.annotated.known.phased.vcf.gz MCF7_ATCC.bam
```

## Supplemental References


Akbari,V. *et al.* (2021) Genome-Wide Detection of Imprinted Differentially Methylated Regions Using Nanopore Sequencing. *bioRxiv*, 2021.07.17.452734.

Buels,R. *et al.* (2016) JBrowse: a dynamic web platform for genome visualization and analysis. *Genome Biol.*, 17, 66.

DePristo,M.A. *et al.* (2011) A framework for variation discovery and genotyping using next-generation DNA sequencing data. *Nat. Genet.*, 43, 491–498.

Dreos,R. *et al.* (2017) The eukaryotic promoter database in its 30th year: focus on non-vertebrate organisms. *Nucleic Acids Res.*, 45, D51–D55.

Li,H. (2018) Minimap2: pairwise alignment for nucleotide sequences. *Bioinformatics*.

Murray,I.A. *et al.* (2018) The non-specific adenine DNA methyltransferase M.EcoGII. *Nucleic Acids Res.*, 46, 840–848.

Patterson,M. *et al.* (2015) WhatsHap: Weighted Haplotype Assembly for Future-Generation Sequencing Reads. *J. Comput. Biol.*, 22, 498–509.

Robinson,J.T. *et al.* (2011) Integrative genomics viewer. *Nat. Biotechnol.*, 29, 24–26.

Shipony,Z. *et al.* (2020) Long-range single-molecule mapping of chromatin accessibility in eukaryotes. *Nat. Methods*, 17, 319–327.
